# Impact of Early Rehabilitation in a Four-Year-Old Patient With Developmental Dysplasia of the Hip: A Case Report

**DOI:** 10.7759/cureus.54900

**Published:** 2024-02-25

**Authors:** Nandini C Baheti, Pratik Phansopkar

**Affiliations:** 1 Musculoskeletal Physiotherapy, Ravi Nair Physiotherapy College, Datta Meghe Institute of Higher Education and Research, Wardha, IND

**Keywords:** perkins line, soft tissue reconstruction, rehabilitation, shenton line, developmental dysplasia of hip

## Abstract

Developmental dysplasia of the hip (DDH) represents a complex spectrum of hip abnormalities, varying from mild dysplasia to severe dislocation, significantly impacting biomechanics and joint stability. This study explores the intricate pathogenesis of DDH, emphasizing its articular and periarticular anatomical anomalies and their profound implications. Factors such as breech positioning, advanced maternal age, postmaturity, and intrauterine crowding contribute to the complexity of DDH's etiology. The fetal development of the hip joint, crucial for understanding DDH, involves intricate processes starting from the fourth week of gestation. Any disruption during this period can lead to abnormal hip development, necessitating early detection and intervention. This is a case presentation of a four-year-old girl with bilateral DDH in detail, highlighting the clinical findings, diagnostic procedures, and physiotherapeutic management employed. A tailored physiotherapy plan was implemented, focusing on pain management, pressure sore prevention, respiratory care, and muscle strength preservation. This study highlights the need for further research in this area by illuminating the complexities of DDH. Despite difficulties and limitations in the literature, interest in researching different facets of DDH is expanding.

## Introduction

Hip subluxation, acetabular dysplasia, genuine hip dislocation, and neonatal instability are all included in the broad category of hip abnormalities referred to as developmental dysplasia of the hip (DDH) [1-3]. Depending on the population under investigation, the screening technique, and the diagnostic criteria employed, the frequency of DDH might range from 1.5 to 25.0 per 1000 live births [4]. When there is dysplasia, articular surfaces are in touch concentrically despite a few morphological abnormalities in the proximal femur, acetabulum, or both. Both articular surfaces make contact in the subluxated hip, although not concentrically. The proximal femur's and acetabulum's articular surfaces do not come into touch during a genuine dislocation [5]. Every hip structure is impacted by a few adaptive changes that occur throughout time and with growth.

A femoral head in concentric contact is important for the formation of the acetabular cavity. The acetabulum cavity flattens and the osseous wall expands if the femoral head is not decreased [6]. Two primary elements are required for the hip to grow and develop maturely, which are the femoral head's concentric location inside the acetabular cavity and the proper growth balance between the acetabular and triradiate cartilage. Hip dysplasia is caused by any modification to these two diseases [7]. Genetic and mechanical factors are among the many complicated underlying reasons. Genetic explanations may be the cause for the higher frequency in female children and those with a positive family history. An increased incidence of breech deliveries, advanced maternal age, postmaturity, oligohydramnios, or any other crowded intrauterine situation are examples of mechanical factors [8]. Compared to the healthy side, there is less abduction in hip dislocations. A newborn's hip abduction ranges from 80 to 90 degrees; any uneven restriction of this range should raise the possibility of a dislocated hip [9]. According to reports, within the first six weeks of life, up to 96% of pathologic abnormalities shown on echography resolve on their own. Thirty-one hips that tested positive for Barlow might be watched for four to six weeks as they naturally stabilize. Hips that test positive for Ortolani or Barlow and do not stabilize on their own after four to six weeks ought to be treated [10]. For the majority of treated newborns, appropriate intervention, such as bracing or casting, can safely avoid the need for reconstructive surgery if DDH is identified early and referred [11].

The development of the hip joint is largely dependent on the complex interaction between the acetabulum and the femur. DDH can result from any disturbance in their balanced relationship throughout fetal development or infancy. The lower limb buds erect during the fourth week of pregnancy. As chondroblasts assemble, the foundation for the hip joint's eventual bones is created. By the sixth week of life, precartilage develops into the developing femoral head, which is identical to the acetabulum, and cartilage becomes the femur's diaphysis. Blastemal cells simultaneously shape the trochanteric projection. The seventh week is a critical time when the interzone distinguishes the sides of the hip joint [12]. The acetabulum develops proximally as a shallow depression at a 65-degree angle; this structure has to deepen to 180 degrees subsequently. The femoral head and articular cartilage develop distally. In the meanwhile, autolysis occurs in the intermediate layer, resulting in the formation of ligamentum teres, synovial membrane, and joint space. It is possible to identify the hip joint by the eleventh week. But, during intrauterine growth, an important difference appears: the acetabulum develops more slowly than the femoral head, which leaves the former with inadequate covering. Any break in their communication triggers aberrant growth. An abnormal connection between the acetabulum and femur is caused by prolonged swaddling in an extreme posture, where the hip is stretched, adducted, and immovable, which prevents the hip from developing normally. Interestingly, the acetabulum keeps growing until a child is five years old. Chronic alterations brought on by persistent misalignment include thickening of the ligamentum teres, hypertrophy of the capsule, and development of a neolimbus, or thicker acetabular border. These changes continue the pattern of aberrant growth by impeding both appropriate contact and the femoral head's ability to move [13].

## Case presentation

Patient information

A four-year-old girl came to Acharya Vinoba Bhave Rural Hospital (AVBRH) with her parents. In the present case, the informant, who is the patient's mother, reported the initial complaint as difficulty in walking since birth. Additionally, the mother observed weakness and inadequate development in the bilateral lower limbs of the patient. The patient is the first-born female child in the family, was delivered via full-term caesarian section, and displayed immediate crying after birth. There is no history of intensive care unit (ICU) admission after birth. The patient came to AVBRH where she underwent closed reduction and hip spica cast application for four weeks as a part of orthopedic treatment. After the removal of the cast, the patient was referred to physiotherapy for further management.

Clinical findings

The patient had an ectomorphic built, was conscious, cooperative, and followed commands. On observation, the patient was seen in a supine lying position. On inspection, the patient has no discernible limb length discrepancy, but evident muscle wasting was observed. No dilated veins were present, and the greater trochanters (GT) aligned at the same level without traction. Palpation reveals no local rise in temperature. Both anterior superior iliac spine (ASIS) levels were symmetrical, with no apparent limb length discrepancy. Tenderness was noted over the right hip, and there was restricted bilateral hip abduction. Despite this restriction, active movements of the ankle and toes were unaffected, and distal circulation remained intact. The range of motion (ROM) of the hip joint is elicited in Table 1. The Barlow and Ortolani tests yielded positive results, which indicated DDH; hence, clinical investigations like X-rays were done to confirm the diagnosis.

**Table 1:** Range of motion of the hip joint

Hip joint	Right	Left
Flexion	0^o^-110^o^	0^o^-110^o^
Extension	0^o^-30^o^	0^o^-30^o^
Abduction	0^o^-45^o^	0^o^​​​​-45^o^
Adduction	45^o^-0^o^​​​​​​​	45^o^-0^o^
Internal rotation	0^o^​​​​​​​-45^o^	0^o^-45^o^
External rotation	0^o^​​​​​​-45^o^	0​​​^o^-45^o^

Clinical investigations

**Figure 1:**
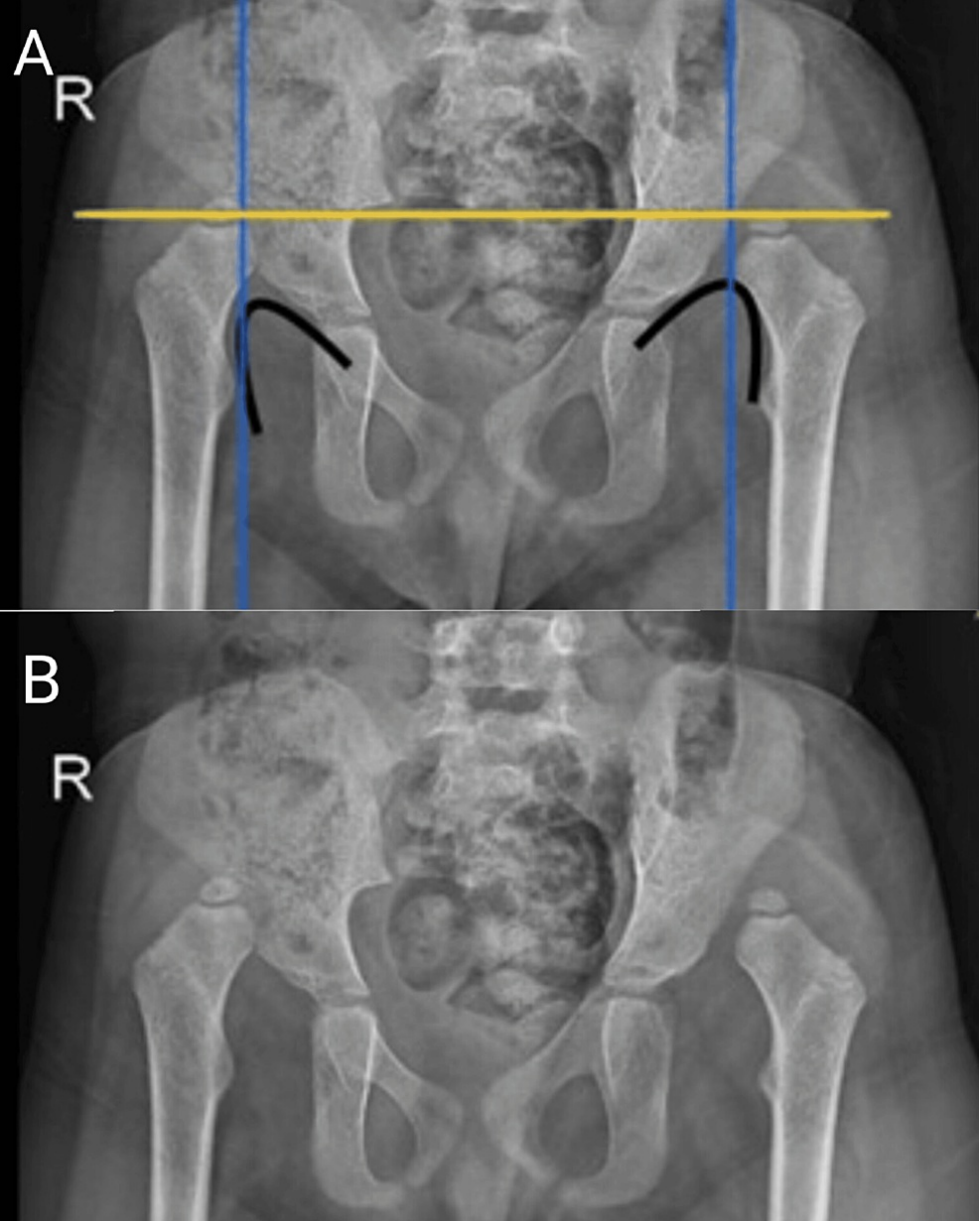
X-ray findings (A) shows a positive Shenton line in black, Perkins line in blue, and Hilgenreiner line in yellow; (B) shows developmental dysplasia of the hip

An X-ray was done showing a positive Shenton line in black, Perkins line in blue, and Hilgenreiner line in yellow, as depicted in Figures 1A-1B. 

Therapeutic intervention

In the patient's care, a four-week protocol was made with each session of 60 minutes, twice a day, with appropriate rest intervals. The treatment protocol is illustrated in Table 2.

**Table 2:** Treatment protocol ROM: range of motion; Reps: repetition; N/A: not applicable

Goal	Intervention	Dosage
To reduce pain	Pain relief techniques (gentle massage, distraction)	10-15 minutes, two to three times a day
To prevent circulatory complications	Ankle pumps	10 reps × 3 sets, three times a day
Prevention of pressure sores	Education on proper positioning to avoid pressure on affected areas. Assistance with repositioning using soft pillows and cushions. Alternating pressure points through regular positional changes	Family education sessions. Regular reinforcement every one to two hours as needed to ensure comfort and relief
To regain ROM of both hip joints	Active assisted ROM exercises for bilateral hip joints in the pain-free range	10 reps × 1 set
Maintaining ROM for all the joints	Active ROM for shoulder, elbow, and wrist joints	10 reps × 1 set
Prevent muscle atrophy and maintain muscle strength	Isometric exercises for the quadriceps, hamstring, and calf muscles. Resistance band exercises for upper limbs. Gradual progression to isotonic exercises	10 reps × 1 set
Provide emotional support and educate the patient and family	Engage in positive communication, addressing concerns and fears. Educate the patient and family about the importance of following the prescribed physiotherapy regimen. Encourage active participation and motivation for rehabilitation	N/A
To develop global motor patterns	Vojta therapy: The Vojta approach focuses on controlling the cervical-thoracic and thoracic-lumbar spine transitions in order to establish a symmetrical shoulder and pelvic girdle position, concentric abdominal muscular activity, autochthonous muscle activity, and appropriate hip joint muscle activity. Reflex crawling is utilized to stimulate activity in the occipital lower limb, proximal attachments, and positioning (penetration) of the hip acetabulum by the femoral head, which places itself in external rotation, abduction, and flexion	5 reps × 1 set

Follow-up and outcome measures

The outcome measures were assessed before and after the rehabilitation program as demonstrated in Table 3.

**Table 3:** Outcome measures

Outcome measures	Pre-rehabilitation	Post-rehabilitation
Visual analogue scale	8/10	3/10
Hip outcome score activities of daily living	20/68	40/68

## Discussion

The majority of the changes leading to a DDH, according to Dunn et al., occur in the latter several months of pregnancy since there is no evidence of hip dysplasia in fetuses that are aborted before 20 weeks of gestation [13]. Many hypotheses and risk factors have been put out to explain the genesis of DDH. The basis of the hormonal theory is an imbalance between progesterone and estrogens. Experimental evidence has shown that estrogens prevent dislocation, but increased progesterone concentrations in the environment can promote dislocation [14]. It is simple to diagnose instability in the newborn period using the Barlow and Ortolani procedures. The Barlow method uses hip adduction and posterior translation to attempt femoral head dislocation; the Ortolani maneuver uses hip abduction and anterior translation to attempt femoral head repositioning [15,16]. A potential bilateral dislocation may be indicated by symmetric restricted abduction, which is abnormal. When a teratologic dislocation occurs, there will be a restricted abduction of the hip and negative instability movements. Leg length disparity (Galeazzi sign) and limb shortening are typically seen with limited abduction [17].

Centric hip reductions are the main goal of therapy because they increase the likelihood of positive functional and anatomical results. Patients with DDH should ideally get treatment for the entirety of their childhood. If this is not possible, the hip joint's intrinsic remodeling potential should be fully utilized by starting treatment as early as possible in childhood, ideally before the age of four [18]. It can be difficult and stressful to raise a child with a medical condition like DDH. We aimed to investigate the whole parenting experience of a kid with DDH. Two motifs were evident. Participants' experiences of losing parental control and needing to rely on others to look after their children were described as giving up parental control. The second topic, battling everyday adjustments, talked about how DDH affected parenting and how individuals struggled to meet their child's expectations. Parenting is impacted by DDH therapy in both practical and emotional ways. The greatest people to help parents of children with DDH are health experts, but before they can do so, they need to understand what the parents need completely [19]. When diagnosing congenital hip joint dysplasia, a physiotherapist who assesses the child's neuromuscular coordination should be included in the diagnostic work-up with a neonatologist and a pediatrician. Vojta suggests using global patterns to cure a hip joint development disorder with a neuromotor etiology. As soon as it is possible, children with congenital hip dysplasia should begin their rehabilitation [20].

## Conclusions

This study sheds light on the intricacies of DDH, emphasizing the necessity of comprehensive research in this field. Despite existing challenges and gaps in the literature, there is a growing interest in exploring various aspects of DDH. Further research endeavors are imperative to enhance our understanding and improve the management of this intricate condition, ensuring optimal outcomes for affected children and their families.
